# Evaluation of a new fusion antigen, cd loop and HAP2-GCS1 domain (cd-HAP) of *Plasmodium falciparum* Generative Cell Specific 1 antigen formulated with various adjuvants, as a transmission blocking vaccine

**DOI:** 10.1186/s12936-023-04798-7

**Published:** 2023-12-09

**Authors:** Zeinab Pourhashem, Leila Nourani, Jafar J. Sani, Hemn Yousefi, Sakineh Pirahmadi, Mobina Sabouri, Abbasali Raz, Navid Dinparast Djadid, Sedigheh Zakeri, Akram Abouie Mehrizi

**Affiliations:** Malaria and Vector Research Group (MVRG), Biotechnology Research Center (BRC), Pasteur Institute of Iran, Tehran, Iran

**Keywords:** Malaria vaccine, Cd-HAP antigen, Adjuvants, Immunogenicity, SMFA, Naturally acquired antibodies

## Abstract

**Background:**

Malaria is a major global health challenge, and for the elimination and eradication of this disease, transmission-blocking vaccines (TBVs) are a priority. *Plasmodium falciparum* Generative Cell Specific 1 (PfGCS1), a promising TBV candidate, is essential for gamete fertilization. The HAP2-GCS1 domain of this antigen as well as its cd loop could induce antibodies that partially inhibit transmission of *P. falciparum.*

**Methods:**

In the current study, a new synthetic fusion antigen containing cd loop and HAP2-GCS1 domain (cd-HAP) of PfGCS1 was evaluated as a transmission blocking vaccine candidate. Initially, the profile of naturally acquired IgG antibodies to the cd-HAP antigen was analysed in Iranian individuals infected with *P. falciparum*, to confirm that this new fusion protein has the appropriate structure containing common epitopes with the native form of PfGCS1. Then, the immunogenicity of cd-HAP was evaluated in BALB/c mice, using different adjuvant systems such as CpG, MPL, QS-21, and a combination of them (CMQ). Furthermore, the blocking efficacy of polyclonal antibodies induced against these formulations was also assessed by oocyst intensity and infection prevalence in the Standard Membrane Feeding Assay (SMFA).

**Results:**

The naturally acquired antibodies (dominantly IgG1 and IgG3 subclasses) induced in *P. falciparum*-infected individuals could recognize the cd-HAP antigen which implies that the new fusion protein has a proper conformation that mimics the native structure of PfGCS1. Concerning the immunogenicity of cd-HAP antigen, the highest IgG levels and titers, by a Th1-type immune profile, and elevated antibody avidity were induced in mice immunized with the cd-HAP antigen formulated with a combination of adjuvants (*P* < 0.0001). Additionally, cytokine profiling of the immunized mice displayed that a high level of IFN-γ response, a Th1-type immune response, was produced by splenocytes from immunized mice that received cd-HAP antigen in combination with CMQ adjuvants (*P* < 0.0001). This formulation of cd-HAP antigen with CMQ adjuvants could reduce oocyst intensity and infection prevalence by 82%, evidenced by the SMFA and hold significant implications for future malaria vaccine development.

**Conclusion:**

Altogether, the results showed that cd-HAP antigen formulated with a combination of the adjuvants (CMQ), could be a promising formulation to develop a PfGCS1-based transmission-blocking vaccine.

**Supplementary Information:**

The online version contains supplementary material available at 10.1186/s12936-023-04798-7.

## Background

Malaria remains a significant public health burden globally, with an estimated 247 million cases and 619,000 deaths reported in 2021 according to the 2022 World Malaria Report of the World Health Organization (WHO) [1, 2]. Despite ongoing efforts to control and eliminate malaria, progress has been uneven, with some regions experiencing frequent cases and deaths in recent years [1]. Strategies for malaria prevention and control, application of insecticide-treated bed nets, indoor residual spraying, and anti-malarial medications encountered challenges such as insecticide and drug resistance, as the significant obstacles to successfully combat malaria [1, 2]. The high burden of malaria and the challenges in controlling the disease highlight the need for effective and safe vaccines to complement existing prevention and control measures and contribute to the long-term reduction, eventual elimination, and eradication of malaria.

Concerning malaria vaccine development, transmission-blocking vaccines (TBVs) play a main role in interrupting malaria transmission [3]. Several TBV candidates have been identified, including those targeting the gametocyte surface protein Pfs48/45 [4], the gametocyte-specific protein Pfs230 [5], the ookinetes surface protein Pfs25 [6], and the gamete fusion protein HAP2 [7]. However, the existing TBV candidates and formulations were not optimal and new candidates or formulations should be evaluated to develop effective TBVs.

Generative Cell Specific 1 (GCS1) or HAP2 antigen is a main TBV candidate antigen, which is present on the surface of male gametocytes and has an essential role in fertilization and is conserved across different *Plasmodium* species [8, 9]. It has been shown that *Plasmodium berghei* GCS1 knockout parasites are unable to undergo fertilization [10, 11]. Structurally, GCS1 (HAP2) is a type 1 transmembrane protein containing N-terminal (extra-cellular) and C-terminal (intra-cellular) parts, and the N-terminal fragment has a conserved HAP2-GCS1 domain [12]. It has been shown that either the full N-terminal or the HAP2-GCS1 domain of the GCS1 antigen are critical to undergo fertilization and deletion of each of them leads to 100% inhibition of gamete fusion and zygote formation [12]. In 2013, Miura et al*.* [13] showed that a partial recombinant protein of the N-terminal of PfGCS1, comprised of the HAP2-GCS1 domain, can induce polyclonal antibodies that partially inhibit oocyst formation in the midgut of *Anopheles stephensi* in standard membrane feeding assay (SMFA). In addition, in 2017, Angrisano et al*.* [14] demonstrated that targeting a conserved fusion loop of HAP2 (cd loop) in the N-terminal inhibits the transmission of *P. berghei* and *P. falciparum* to *Anopheles*. Therefore, to have the properties of both the cd loop and HAP2-GCS1 domain, a new fusion protein containing both of them (named: cd-HAP) was designed, as a TBV candidate.

Since the recombinant antigens have poor immunogenicity, to improve the immune responses, different strategies including the addition of adjuvants to vaccine formulations can be used [15, 16]. Adjuvants are essential components of vaccines that can enhance the immunogenicity of antigens and promote protective immune responses [17]. Several types of adjuvants have been extensively studied in preclinical and clinical settings, including CpG, MPL, and QS-21, either alone or in combination [15, 16, 18, 19]. In the context of malaria vaccine development, these adjuvants have been used to enhance the immunogenicity of various antigens and vaccine platforms, including subunit vaccines and viral vectors [20]. Given their proven track record of enhancing vaccine immunogenicity, these adjuvants represent promising tools for improving the efficacy of malaria vaccines [21, 22]. Therefore, in the current study, the immunogenicity of the cd-HAP fusion protein was evaluated as a TBV candidate with formulations containing different adjuvants.

In addition, concerning the development of TBVs, it is crucial to gather data on the naturally acquired immunity against the candidate antigens. Recognition of the target antigen by the naturally acquired antibodies could confirm that the structure of the recombinant antigen resembles the native form of antigen with common epitopes. On the other hand, by measuring the levels of different IgG subclasses in response to a candidate antigen, researchers can gain insight into the type of immune response induced by natural infection. IgG antibodies are the primary mediators of naturally acquired immunity to malaria, and different subclasses of IgG antibodies may have different effector functions against the disease. This information can be used to guide the development and optimization of vaccines based on the candidate antigen. In this regard, evaluating the naturally acquired immunity levels against the candidate antigens in malaria patients is suggested. The data can provide valuable insights into the immune response induced by the vaccine and its potential efficacy in blocking malaria transmission, ultimately paving the way for more effective malaria control and eradication strategies [23].

In this study, in line with WHO recommendations, to develop the TBVs needed to eliminate and eradicate malaria, a new fusion antigen was designed and evaluated. Initially, the naturally acquired antibodies raised to cd-HAP antigen were evaluated in Iranian *P. falciparum-*infected individuals to investigate if the new fusion protein has the appropriate structure containing common epitopes with the native form of PfGCS1. Then, the vaccine efficacy of the cd-HAP antigen was evaluated by using the different formulations of adjuvants. Mouse groups were immunized with cd-HAP antigen alone or formulated with CpG, MPL, QS-21, or a combination of three adjuvants (CMQ), and the humoral and cellular immune responses were evaluated. The persistence of immune responses to this fusion antigen was also assessed 6 months after the last immunization. Moreover, the functional activity of the induced antibodies to cd-HAP antigen in mice was assessed using the SMFA. The results of the current study have important implications for the design and optimization of the *P. falciparum* cd-HAP-based malaria vaccine and may ultimately lead to the development of more effective tools for controlling the spread of this devastating disease.

## Methods

### Expression and purification of cd-HAP protein

To produce the recombinant cd-HAP fusion antigen, a new genetic construct containing cd loop and HAP2-GCS1 domain of PfGCS1 was designed. To design this construct, the cd loop (nt: 675–730; aa: 176–195) was fused to nucleotides 1077–1973 (aa: 311–609) of PfGCS1 antigen (accessible in PlasmoDB at Pf3D7_1014200), containing the HAP2-GCS1 domain. The designed sequence was codon-optimized and synthesized by Biomatik Company. This construct was cloned in BamHI/XhoI restriction sites of pET23a to express the cd-HAP antigen with a C-terminal His-tag. To express the cd-HAP antigen, *Escherichia coli* BL21(DE3) cells harbouring the pET23a-cd-HAP plasmid were cultured in 0.5 × LB medium at 37 °C and induced with IPTG at a concentration of 0.5 mM after the turbidity absorbance of the medium reached λ600nm = 0.8 (OD). After 18 h of induction, cells were harvested via centrifugation at 7000 rpm, for 15 min. The resulting cell pellet containing inclusion bodies with recombinant cd-HAP was solubilized in a lysis buffer (1% Triton X, 50 mM Tris–HCl, and 500 mM NaCl, pH:7). The pellet was then sonicated for 10 min and the insoluble fraction was washed twice with 1 × phosphate-buffered saline (PBS) before being re-suspended in the second lysis buffer (8 M urea, 20 mM Tris–HCl, 500 mM NaCl, 20 mM Imidazole, 5% Glycerol, 0.5% Tween and 10 mM β-mercaptoethanol at pH:7.5). The re-suspended mixture was then incubated at 4 °C for 90 min with gentle agitation. Following another round of sonication and centrifugation at 7000 rpm for 15 min, the supernatant was collected and incubated with 500 µl Ni–NTA agarose (Qiagen, Hilden, Germany) at 4 °C for 90 min with gentle agitation. Ni–NTA resin was washed with a 100 ml buffer containing 4 M urea, 20 mM Tris–HCl, 750 mM NaCl, 40 mM Imidazole and 10 mM β-mercaptoethanol (pH 7.5). The recombinant protein was eluted using elution buffer containing: 2 M urea, 20 mM Tris–HCl, 300 mM NaCl, 250 mM Imidazole, and 10 mM β-mercaptoethanol (pH:7.2). The purified protein was subjected to step-wise dialysis against a buffer containing 1 M urea, 300 mM NaCl, and 5 mM Immidazol overnight, then 2 h against a buffer containing 300 mM NaCl, and 5 mM Immidazol using a 14-kDa cut-off dialysis bags, after which the desalted protein was analysed on SDS-PAGE, and confirmed by Western blotting with an anti-His antibody (Penta-His Antibody; Qiagen, Germany). The purified protein was quantified using a calorimetric Bradford assay. Lastly, the *E. coli* endotoxin level was determined using the LAL kit PYROSTAR^™^ ES-F, FUJIFILM, USA).

### Ethical considerations

The study subjects included 37 *P. falciparum*-infected individuals in Chabahar, Sistan and Baluchistan Province in south-eastern Iran (2005–2010), who were confirmed to have anti-PfGCS1 IgG antibodies [24]. All participants provided written informed consent before enrolling in the study, approved by the Ethical Review Committee of Research of the Pasteur Institute of Iran [IR.PII.REC.1399.071]. Blood samples were collected from all participants at admission, and sera were separated by centrifugation and stored at − 20 °C until further analysis.

### Assessment of naturally acquired anti-cd-HAP antibodies in *P. falciparum* infected individuals using ELISA

In this study, the IgG and IgG subclass antibodies to cd-HAP were evaluated in patients infected with *P. falciparum* (n = 37) that have been confirmed to have anti-PfGCS1 (aa: 311–609 of PfGCS1) IgG antibodies [24] using an indirect enzyme-linked immunosorbent assay (ELISA). To prepare the cd-HAP antigen, the recombinant cd-HAP antigen (aa: 176–195 (cd loop) fused to aa: 311–609 of PfGCS1 antigen containing HAP2-GCS1 domain) was expressed in pET23a-*E. coli* BL21(DE3) and purified using Ni–NTA agarose. The ELISA was optimized by performing a checkerboard cross-titration assay with varying antigen concentrations (10–2000 ng) and antibody dilutions (1:100–1:3200). MaxiSorp flat-bottom 96-well ELISA plates were coated with cd-HAP antigen at optimized concentration 1000 ng/ml and incubated overnight at 4 °C. After blocking with 2% BSA, serum samples were added at a dilution of 1:600. Anti-human IgG antibody conjugated with horseradish peroxidase (HRP) (Sigma-Aldrich, USA) was added at 1:25,000 dilution. To determine the IgG1, IgG2, IgG3, and IgG4 subclass antibody responses to cd-HAP, biotin-conjugated isotype-specific anti-human IgG subclass antibodies (Sigma-Aldrich Co.) were used at a dilution of 1:2500, followed by streptavidin-conjugated antibody at a concentration of 1:2500 in PBS. TMB (3,3′,5,5′-Tetramethylbenzidine) was used as the substrate for detection, and the reaction was stopped with 2N H_2_SO_4_. OD_450_nm was measured using an ELISA microplate reader (BioTek, Winooski, VT, USA). To establish negative controls, plasma samples from 15 healthy individuals who had not been exposed to malaria and were from non-endemic regions were used. The threshold for positivity (cut-off value) was determined by calculating the mean optical density (OD) of the negative samples plus three standard deviations (SD). Any sample with an OD value higher than the cut-off value was considered a positive responder with anti-cd-HAP IgG subclass antibodies.

### Immunization of BALB⁄c mice with cd-HAP antigen in different formulations

Female BALB/c mice were used to generate polyclonal antibodies against cd-HAP. The mice, aged 6–8 weeks and inbred, were randomly divided into 10 groups (n = 9/group, Table 1) and immunized subcutaneously at the base of the tail with cd-HAP (10 μg/mouse/prime and 5 μg/mouse/boost) either alone (Group 1: non-adjuvanted immunized mouse group) or formulated with CpG (10 μg/mouse, vaccine grade type, InvivoGen, San Diego, CA, USA, Group 2), MPL (10 μg/mouse, vaccine grade type, InvivoGen, San Diego, CA, USA, Group 3), QS-21 (10 μg/mouse, vaccine grade type, Alfa Diagnostic Cat No: AV-4010, Group 4), or cd-HAP antigen in combination with 5 μg/mouse CpG, 5 μg/mouse MPL, and 5 μg/mouse QS-21 adjuvants in Group 5, as adjuvanted immunized groups, three times at 14-day intervals. The control groups G6-10 received 1 × PBS alone or each of single adjuvants (G7-G9) or a mixture of three adjuvants (G10). The procedures involving animals were approved by the Committee of Animal Ethics of the Pasteur Institute of Iran (IR.PII.REC.1399.080). Sera were collected from the mice before the first injection [as pre-immune sera/normal mouse sera (NMS)] and on days 10, 24, 38, and 180 after the primary immunization (Table 1) to evaluate anti-cd-HAP antibody responses. The collected serum samples were stored at − 20 °C.

**Table 1:** Experimental groups and immunization protocol

Group no	Antigen/Adjuvant(s)	Ag (μg/mouse)			Adjuvants (μg/mouse)		
		Prime*	Boost1**	Boost 2***	CpG	MPL	QS-21
1 (n = 9)	cd-HAP/−	10	5	5	–	–	–
2 (n = 9)	cd-HAP/CpG	10	5	5	10	–	–
3 (n = 9)	cd-HAP/MPL	10	5	5	–	10	–
4 (n = 9)	cd-HAP/QS-21	10	5	5	–	–	10
5 (n = 9)	cd-HAP/CpG + MPL + QS-21	10	5	5	5	5	5
6 (n = 9)	−/1 × PBS	–	–	–			
7 (n = 9)	−/CpG	–	–	–	10	–	–
8 (n = 9)	−/MPL	–	–	–	–	10	–
9 (n = 9)	−/QS-21	–	–	–	–	–	10
10 (n = 9)	−/CpG + MPL + QS-21	–	–	–	5	5	5

Female BALB/c mice (n = 90) were distributed into 10 groups and immunized subcutaneously with the antigen alone or in combination with different adjuvants. The control groups received 1 × PBS alone or with each adjuvant. Sera samples were collected from the tail vein on days 10, 24, 38, and 180 after the first immunization for further analysis

^*^Day 0

^**^Day 14

^***^Day 28

### Quantification of anti cd-HAP antibody responses using ELISA in immunized BALB/c mice

Anti-cd-HAP IgG in the serum of different mouse groups from pre-immunization, days 10, 24, 38 (n = 9 mouse sera in each group in mentioned time points) and 180 (n = 4 mouse sera in each group) after primary immunization were evaluated using ELISA. Optimized antigen concentrations and the dilution of the primary and secondary antibodies were evaluated by a checkerboard cross-titration assay using different concentrations of antigen and antibody dilutions. The optimized concentration of cd-HAP (1000 ng/ml, 100 ng/well) was added into MaxiSorp flat-bottom 96-well ELISA plates (Jet Biofil, Guangzhou, China) and incubated at 4 °C overnight. After blocking, optimized dilution of serum samples (1:200) was added to the desired wells. After incubation and rinsing of the wells, a secondary HRP-conjugated anti-mouse IgG antibody at 1:25,000 dilution (Sigma-Aldrich Co., USA) was added. Anti-cd-HAP IgG was detected using TMB as a substrate. The reaction was stopped with 2N H_2_SO_4_, and the absorbance was measured using an ELISA microplate reader (BioTek, Winooski, VT, USA) at OD_450_nm. The cut-off values were estimated from the average of the 20 NMS plus three standard deviations (SD). Further, the anti-cd-HAP IgG subclasses (IgG1, IgG2a, IgG2b and IgG3) were evaluated by the ELISA as described above; however, the secondary antibodies specific to mouse IgG1 and IgG3 at 1:2000 dilution, and IgG2a, and IgG2b at 1: 1000 dilution of antibodies were used (Sigma-Aldrich Co.). Subsequently, the plates were incubated with 1:10,000 dilution of anti-goat IgG HRP (Sigma-Aldrich Co., USA) at RT for 1 h. For analysis of the antibodies persistence, the levels of antibodies on day 180 (6 months after immunization) was measured and compared with day 38 (10 days after the second boost) using paired sample t-test.

### Anti- cd-HAP antibody titration and avidity in immunized mice

Titration assay is a common technique used to determine the concentration of a substance in a solution. To perform an antibody titration assay of anti-cd-HAP IgG and its subclasses (IgG1, IgG2a, IgG2b, and IgG3), the ELISA was performed as described above. Briefly, after antigen coating (100 ng/well), a serial dilution (ranging from 1:200 to 1: 409,600) of pooled mouse sera (n = 9) from each group obtained on day 38 after the first immunization was prepared. 100 µl of diluted antibodies were then added to the corresponding wells in a microplate, followed by washing to remove any unbound antibodies. The secondary anti-mouse IgG antibody, conjugated with a peroxidase enzyme, was then added to the wells (at 1:25,000 dilution), and the plate was incubated for 1 h, at RT. Besides, regarding IgG subclasses, after incubation with serial dilutions of pooled sera, the secondary antibodies specific to mouse IgG1 and IgG3 at 1:2000 dilution, and IgG2a, and IgG2b at 1: 1000 dilution of antibodies were used (Sigma-Aldrich Co.) and incubated for 1 h, at RT. After the washing step, anti-goat IgG HRP (Sigma-Aldrich Co.) with 1:10,000 dilution was added and incubated for 1 h, at RT. After a final wash step, the TMB substrate solution was added, which reacted with the conjugated enzyme to produce a detectable signal. The signal intensity was measured using an ELISA microplate reader, and the endpoint titer of sera was determined by the dilution of the primary antibody that produced an OD value above the cut-off, which is estimated from the average of the 20 NMS plus three standard deviations (SD).

To assess the anti-cd-HAP IgG avidity and its subclasses, a modified ELISA was conducted according to the published procedures [25, 26]. Dilutions of individual mouse sera (n = 9) from each group, obtained on day 38 after the first immunization (1:200), were added to two separate microplates, each containing the optimal concentration of antigen (100 ng/well). The ELISA procedure was carried out as described above, but after serum incubation, one plate was washed three times with PBS-T, and the other was washed with buffer containing PBS-T and 5 M urea. The avidity index was measured after subtracting the OD of NMS (background) with the following formula: AI = [(OD_450_ of the sample with urea)−(OD_450_ of NMS with urea)]/[(OD_450_ of the sample without urea)−(OD_450_ of NMS without urea)] × 100. Low-, intermediate-, and high-avidity anti-cd-HAP-specific antibodies were determined as AI values of < 30%, 30% to 50%, and > 50%, respectively [27].

### Cytokine assay

Cytokine profiles, including IFN-γ, TNF, IL-4, and IL-10, were measured and analysed using ELISA kits (R&D Systems, Minneapolis, USA) from supernatants of splenocytes stimulated with cd-HAP in immunized mouse groups on days 38 and 180 after primary immunization. Single-cell suspensions of splenocytes from each mouse group (3 randomly selected mice in each group) were prepared in RPMI 1640 medium (Gibco, Invitrogen, Scotland, UK), and RBCs were removed by ammonium chloride-potassium lysis buffer (pH 7.2). The splenocytes re-suspended in RPMI 1640 medium containing 5% FCS (Fetal Calf Serum, Sigma-Aldrich Co.), 10 mM HEPES (Sigma-Aldrich Co.), penicillin–streptomycin (100 U-100 µg/ml), and 2.3 × 10^–2^ mM 2-mercaptoethanol (2-ME). 100 µl of cell suspension (3 × 10^6^ cells/ml, 3 × 10^5^ cells/well) were cultured in a flat-bottom 96-well tissue culture plate (Orange Scientific, EU, Belgium) in three replicates in the presence of 100 µl of ConA (5 µg/ml) as the positive control, medium alone as the negative control and cd-HAP antigen (4 µg/ml, optimized concentration). The plate was then incubated in a 5% CO_2_ incubator in a humidified atmosphere at 37 °C, and supernatants were collected after 24 h for IL-4, 72 h for IL-10 and TNF and 120 h later for IFN-γ measurement and stored at − 70 °C. 100 µL of supernatants were used to measure each cytokine using ELISA kits (R&D Systems, Minneapolis, USA). The concentration of cytokines was calculated based on the standard curves performed in parallel with known concentrations of recombinant mouse IL-4, IL-10, TNF, and IFN-γ for each experiment. For this purpose, 100 µl of the collected supernatants of spleen lymphocytes after culture in the presence of the optimum concentration of cd-HAP (4 µg/ml, 400 ng/well) were analysed using the ELISA method and kit instructions. To remove the backgrounds, the concentration of each cytokine in the negative control (by media) was subtracted from the concentration of the same cytokine of the test group stimulated by antigen (delta IL-4, IL-10, TNF, and IFN-γ). The mean concentration ± SD was recorded for each set of tested samples. IL-4 (humoral immunity index) and IFN-γ (cellular immunity index) were used as indicators of Th2 and Th1 type of immune responses, respectively, while IL-10 and TNF were used to assess the overall immune regulation.

### Standard membrane feeding assay

In order to evaluate the inhibitory effects of induced antibodies on oocyst formation, the continuous culture of *P. falciparum* was used to produce gametocytes in vitro. The gametocytes were produced from the NF54 strain of *P. falciparum*, generously provided by the Pasteur Institute of Paris, and were continuously cultured in RPMI 1640 medium supplemented with 5% pooled human AB^+^ serum, 0.2% (wt/vol) AlbuMAX II, 25 mM HEPES, 50 mg/litre of hypoxanthine, 1.96 g/liter of glucose, and 25 mM NaHCO_3_ at pH 7.2, all of which were optimized for gametocyte production. The gametocyte induction protocol established by Fivelman et al*.* [28] was followed, and the gametocytes were classified into different stages based on the Carter and Miller guidelines [29]. Mature gametocytes with a male: female ratio of 1: 2–4 were expected to be harvested 12 days after initiation, and their maturity was confirmed using the exflagellation test. The *An. stephensi* mosquitoes were maintained in the National Insectarium of Iran, Karaj site under controlled conditions of 26 °C temperature and 80% humidity with a 12/12 light/dark cycle. The standard membrane feeding assay (SMFA) technique was used to evaluate the inhibitory effects of antibodies on oocyst formation. Briefly, 4–5-day-old female mosquitoes (≥ 50 per feeding box) were allowed to feed on blood infected with gametocytes and serum from different immunized groups for 30 min. For feeding female *An. stephensi*, mature stage of *P. falciparum* NF54 gametocytes were mixed with washed human O + blood group at 50% hematocrit in human AB + serum, and an equal amount of mouse pooled sera from test or control groups were added to the mixture. Pooled pre-immune mice sera (n = 30) was utilized in a control group as normal mouse sera (NMS). Fully fed mosquitoes were selected, and their mid-guts were dissected and stained using 0.2% mercurochrome in 1 × PBS on days 8–10 post-feeding. The stained oocysts were counted under a light microscope after staining with 0.2% mercurochrome diluted in PBS for 10 min. Two independent repeats were performed and the percentage of inhibition in mean oocyst count per mosquito (transmission reducing activity, TRA) was estimated as follows: 100 × [1−(mean number of oocyst in the test group/mean number of oocyst in the control group)]. In addition, the percentage inhibition in the prevalence of infected mosquitoes (transmission-blocking activity, TBA) was calculated as follows: 100 × [1−(proportion of mosquitoes with any oocysts in the test group/proportion of mosquitoes with any oocysts in the control group)].

### Statistical analysis

The normality of the data obtained for both humoral and cellular responses was confirmed using the Shapiro–Wilk test. Given the normal distribution of the data, differences in antibody levels and cytokine responses among all mouse groups were evaluated using One-way ANOVA. To account for multiple comparisons following ANOVA, the Bonferroni post hoc test was employed for inter-group comparison. Furthermore, a paired sample t-test was applied to compare antibody levels within each group at different time points (on days 10, 24, 38, and 180) and Bonferroni adjusted P values were reported. P < 0.05 was considered statistically significant.

Regarding SMFA analysis, the Shapiro–Wilk test was performed and the results showed that the data did not follow normal distribution. Therefore, the Kruskal–Wallis H test was employed to evaluate differences in the intensity of oocyst production across different groups. Following the Kruskal–Wallis H test, pairwise comparisons were performed using the Mann–Whitney U test to further investigate differences in oocyst production intensity between test and control groups. In addition, Fisher’s exact test was utilized to examine differences in the frequency of mosquito infection across the groups. All P values were adjusted with Bonferroni correction analysis and the final P values were presented. A confidence level of P < 0.05 was considered statistically significant.

## Results

### Expression and purification of antigen

**Fig. 1:**
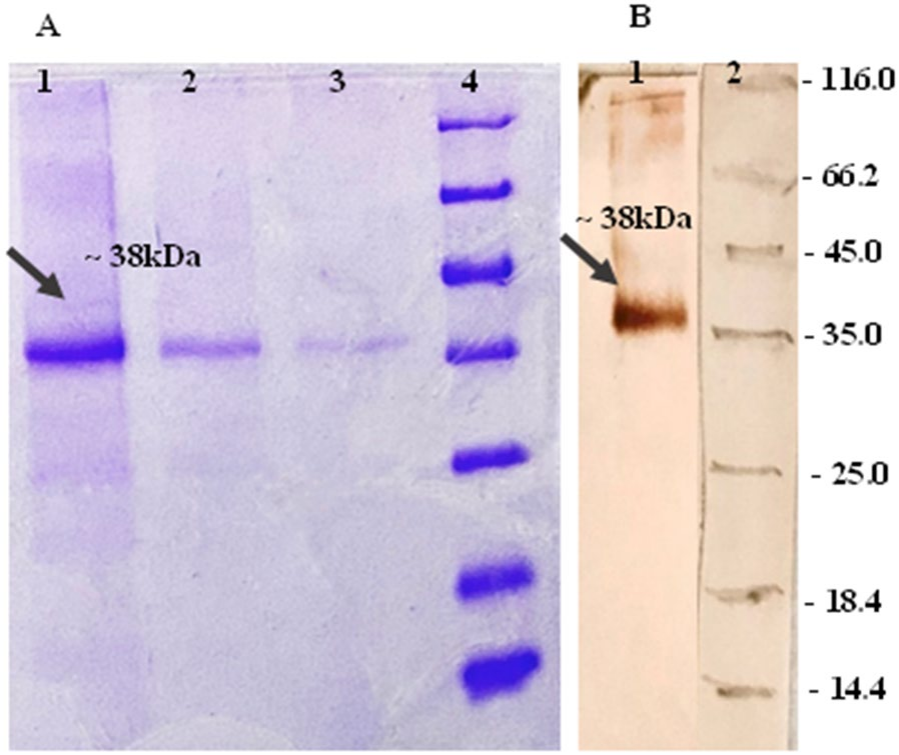
SDS-PAGE and Western blot analysis of cd-HAP. **A** SDS-PAGE analysis of purified cd-HAP (Lanes 1–3), and lane 4: molecular weight protein marker (Fermentas, 116–14.4 kDa). **B** Western blot analysis of cd-HAP protein with anti-His tag mAb (Lane1) and lane 2: molecular weight protein marker (Fermentas, 116–14.4 kDa)

In this study, the expression and purification of cd-HAP antigen were carried out using the pET23a-cd-HAP construct in *E. coli* BL21(DE3) cells. Following induction with IPTG, the cd-HAP protein was purified and analysed by SDS-PAGE, revealing a single band at the expected molecular weight of 38 kDa confirmed by western blotting using a His-tag-specific antibody, which detected the protein at 16 h after induction. These results demonstrate successful expression and purification of cd-HAP antigen, which can be used for further immunological studies (Fig. 1).

### Confirming the presence of native epitopes on the cd-HAP by *P. falciparum* infected sera

**Fig. 2:**
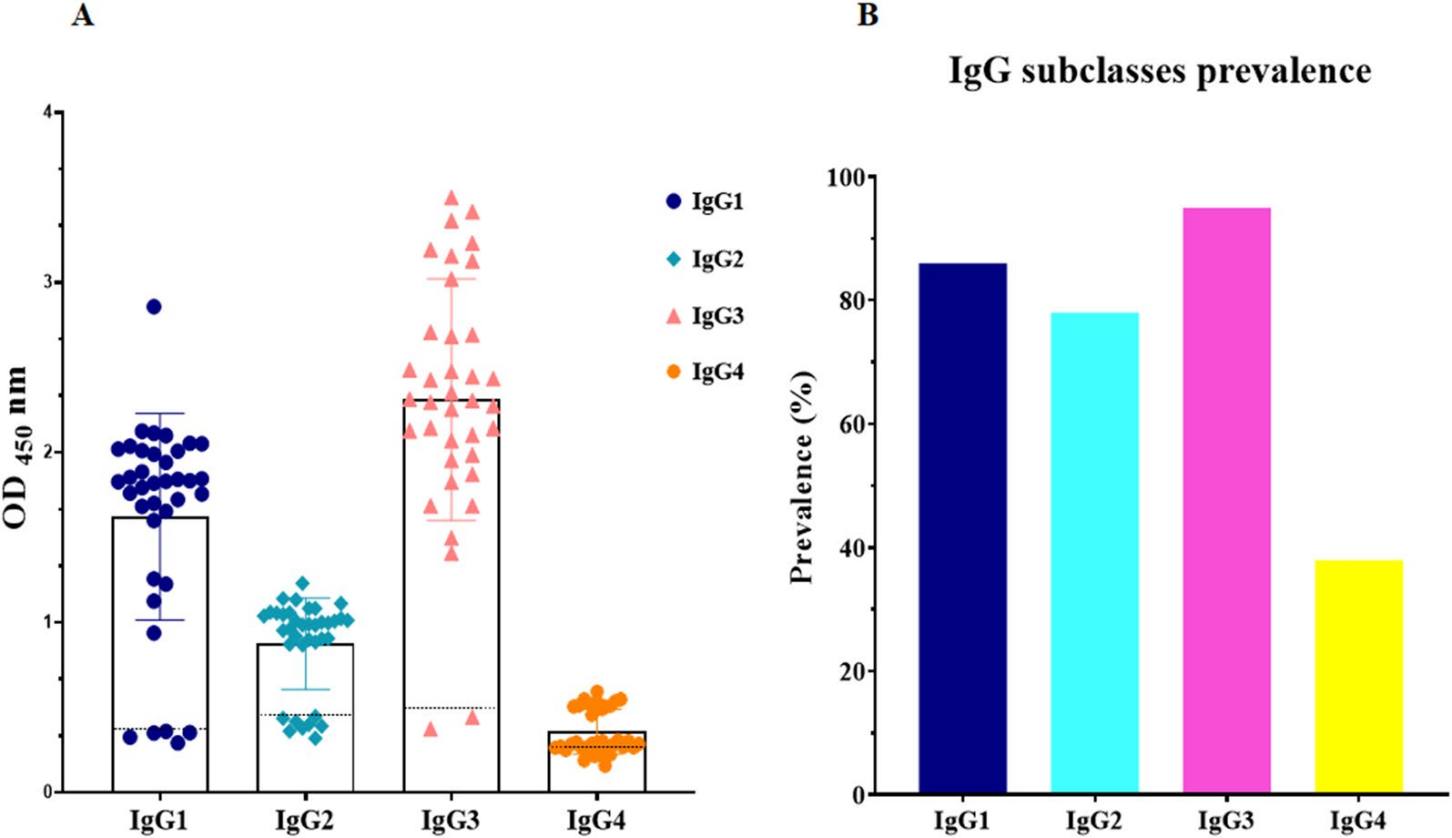
Analysis of cd-HAP-specific IgG subclasses antibody responses in *P. falciparum*-infected patients in malaria endemic area from Iran. **A** Optical density (OD) values for IgG subclasses in 37 serum samples positive for anti-cd-HAP IgG. IgG3 was the most prevalent subclass, followed by IgG1, IgG2, and IgG4. **B** Prevalence of IgG subclasses in the same samples, showing that IgG3 and IgG1 were the most prevalent subclasses

Among tested sera from *P. falciparum* malaria patients, all 37 serum samples were able to recognize the cd-HAP antigen (Fig. 2). Analysis of anti-cd-HAP IgG subclasses revealed that IgG3 was the predominant subclass, present in 35 samples (95%, mean OD_450_nm: 2.3), followed by IgG1, present in 32 samples (86%, mean OD_450_nm: 1.821). IgG2 was detected in 29 samples (78%, mean OD_450_nm: 1.00), and IgG4 existed in 14 samples (38%, mean OD_450_nm: 0.519).

### Evaluation of the anti cd-HAP IgG levels and its subclasses in immunized BALB/c mice

**Fig. 3:**
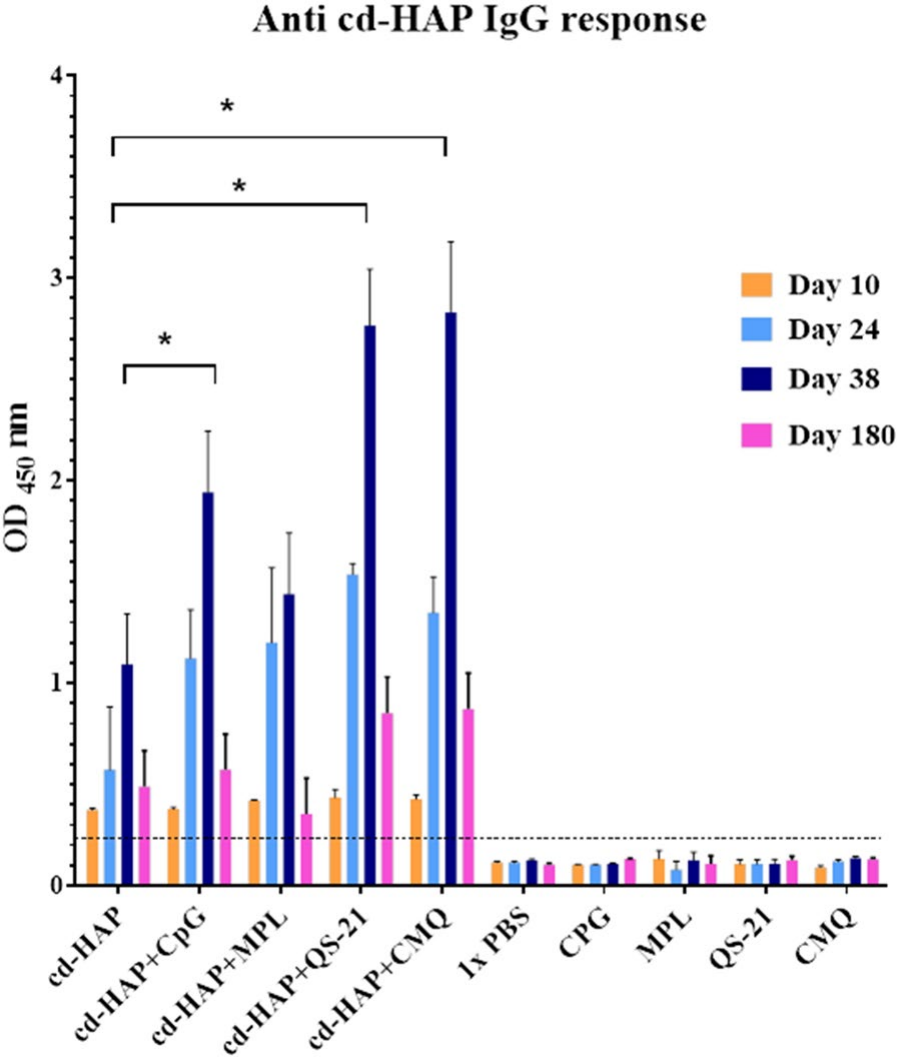
Evaluation of anti-cd-HAP IgG antibody levels in vaccinated mouse groups. The mouse groups were immunized subcutaneously with recombinant cd-HAP alone or in combination with different adjuvants, including CpG, MPL, QS-21, and CMQ (CpG/MPL/QS-21), while control groups received 1 × PBS, CPG (10 µg), MPL (10 µg), QS-21(10 µg) or CMQ mixture (5 µg of each adjuvant). Anti-cd-HAP IgG antibody levels were measured at various time points, including days 10, 24, 38, and 180, after the first immunization in each mouse group using ELISA. The total IgG antibody levels showed significant differences between different immunization time points in each vaccine group (P < 0.05, paired-sample t-test). On day 38, the anti-cd-HAP IgG antibody levels among the vaccinated mouse groups were significantly different (P < 0.0001, One-way ANOVA test), with the highest level observed in group receiving cd-HAP in combination with CMQ or QS-21 adjuvants. Multiple comparison analysis was used to identify the statistical differences between anti-cd-HAP IgG antibodies among groups on day 38 (Bonferroni post hoc test, *P < 0.0001). The bars and error bars represent the mean OD_450_ values and standard deviations (SD) for individual mice in each group, respectively

**Fig. 4:**
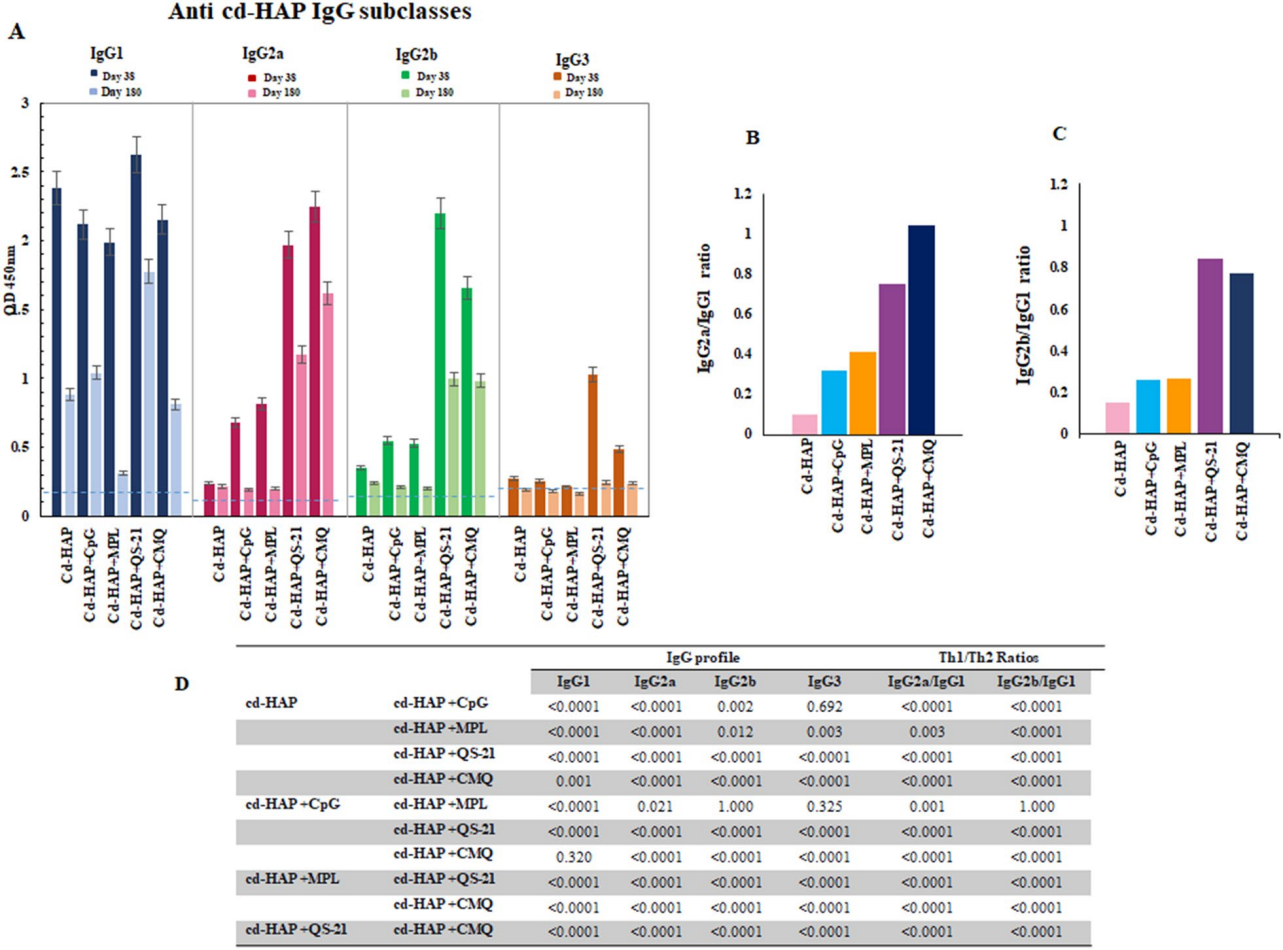
Analysis of anti-cd-HAP IgG subclasses profiles, persistence and evaluation the Th1/Th2 ratios. **A** The profile of anti- cd-HAP IgG subclasses among vaccinated mouse groups for 9 individual mice (day 38) and 3 individual mice (day 180) in each group, respectively. The bars and error bars show the mean OD_450_ values and standard deviations (SD). **B**, **C** the Th1/Th2 ratio was calculated for each group on day 38 after the first immunization. One-way ANOVA analysis showed significant mean difference for the level of anti-cd-HAP IgG1, IgG2a, IgG2b, and IgG3 antibodies between vaccine groups 1–5 (P < 0.0001). **D** The Table shows multiple comparisons of means performed by Bonferroni post hoc test for anti-cd-HAP IgG subclasses among vaccine groups on day 38 after primary immunization

To evaluate the immunogenicity of cd-HAP antigen formulated with different adjuvants in BALB/c mice, following a two-boost regimen, the levels of anti-cd-HAP IgG antibodies were measured using ELISA. The results demonstrated a significant increase in the levels of anti-cd-HAP IgG antibodies on days 24 and 38, compared to day 10 after primary immunization, in all immunized mouse groups receiving cd-HAP antigen alone or formulated with adjuvants (P < 0.0001 by a paired-sample t-test). The groups that received cd-HAP formulated with three adjuvants (CMQ, group 5) and QS-21 (Group 4) exhibited the highest levels of anti-cd-HAP IgG antibodies (mean OD_450_nm = 2.83 and 2.76, respectively), while the group that received cd-HAP alone had the lowest level (mean OD_450_nm = 1.09) (P < 0.0001, One-way ANOVA, Fig. 3). Among mice immunized with cd-HAP formulated with individual adjuvants, Group 4 (cd-HAP /QS-21) indicated a significant increase in the level of anti-cd-HAP IgG compared to Group 2 (cd-HAP/CpG) and Group 3 (cd-HAP/MPL) on day 38 of the first immunization (P < 0.0001, Bonferroni post hoc test). The data derived from multiple comparisons of means, conducted using the Bonferroni post hoc test for anti-cd-HAP IgG subclasses among vaccine groups on day 38 after primary immunization, are detailed in Fig. 4D. Among adjuvanted groups, on day 180, the level of anti-cd-HAP IgG antibodies decreased in all groups relative to day 38 after the first immunization. (P < 0.05 by a paired-sample t-test; Fig. 3). However, groups 4 and 5 receiving cd-HAP antigen with QS-21 and CMQ, respectively, had still higher levels of anti-cd-HAP IgG antibodies relative to other immunized groups on day 180 (P < 0.0001, One-way ANOVA, Fig. 3).

The anti-cd-HAP IgG subclasses in sera from immunized mouse groups were analysed at two distinct time points: 38 days and 180 days after primary immunization. On day 38, Group 5 (cd-HAP/CMQ) showed the highest level of anti-cd-HAP IgG2a with a mean OD_450_nm of 2.25. However, Group 4 (cd-HAP/QS-21) had the highest levels of IgG1 and IgG2b with mean OD_450_nm values of 2.071 and 2.2, respectively. The lowest level of IgG1 was found in Group 3 (cd-HAP/MPL) with a mean OD_450_nm of 0.564. Group 1 (cd-HAP antigen alone) showed the lowest levels of IgG2a and IgG2b antibodies with mean OD_450_nm values of 0.237 and 0.348, respectively (P < 0.001, One-way ANOVA, Fig. 4A).

On day 180, all vaccinated mouse groups showed a significant decrease in subclass levels compared to day 38. Among these groups, Group 4 (cd-HAP/QS-21) showed the least reduction in IgG1 (32% reduction), while Group 5 (cd-HAP/CMQ) showed the least reduction in both IgG2a (28% reduction) and IgG2b (40% reduction) (P < 0.001, One-way ANOVA, Fig. 4A).

To assess the type of antibody response, the Th1/Th2 Ratio (the IgG2a/IgG1 and the IgG2b/IgG1) was measured in immunized mouse groups (Fig. 4B, C). The Th1/Th2 ratio (IgG2a/IgG1 and IgG2b/IgG1) shows which type of immune response has been induced by the vaccine formulations [30, 31]. The results demonstrated a significant variation in the ratios of examined mouse groups (P < 0.0001, One-way ANOVA). The mouse group receiving the cd-HAP antigen along with the combination adjuvants (CMQ) showed the highest IgG2a/IgG1 ratio (1.62; P < 0.05) when compared to other immunized mouse groups on day 38 (P < 0.05, Bonferroni post hoc test) (Fig. 4B). On the other hand, the highest and comparable IgG2b/IgG1 ratio was observed in the mouse groups that received cd-HAP plus QS-21 or CMQ (Fig. 4C).

### Adjuvant formulation enhances avidity of anti-cd-HAP IgG antibodies in immunized mice

In this experiment, mice receiving cd-HAP antigen with different formulations displayed low- or intermediate-avidity anti-cd-HAP IgG antibodies (Table 2). Multiple comparisons of the antibody avidity index (AI) among the adjuvanted immunized groups 2–5 showed that the avidity of anti-cd-HAP IgG antibodies in mouse group 5 (cd-HAP/CMQ) was significantly higher than those in mouse groups 2 and 3 (cd-HAP /MPL and cd-HAP /CpG) on day 38 after the first immunization (P < 0.0001, Bonferroni post hoc test). All immunized groups elicited high avidity IgG1 antibodies. Mouse Groups 4 and 5 receiving cd-HAP with QS-21 and CMQ adjuvants, respectively exhibited high avidity IgG2a antibodies. Mouse Group 5 received cd-HAP with CMQ adjuvants and exhibited the highest AI for the IgG2b subclass. The highest AI for the IgG, IgG2a, and IgG2b subclass antibodies was observed in mouse group 5, which received cd-HAP in the CMQ adjuvants. However, none of the immunized groups had high-avidity IgG, IgG2b, or IgG3 antibodies.

**Table 2:** Mean Avidity indices of elicited anti-cd-HAP IgG, IgG1, IgG2a, and IgG2b antibodies in sera from each immunized mouse group (n = 9 in each group)

	Total IgG (%)	IgG1 (%)	IgG2a (%)	IgG2b (%)	IgG3 (%)
cd-HAP	21	56	25	24	7
cd-HAP + CpG	22	68	32	30	31
cd-HAP + MPL	15	63	18	21	11
cd-HAP + QS-21	44	85	55	40	43
cd-HAP + CMQ	49	82	60	44	42

The AI was determined by dividing the OD value of urea-treated serum samples by that of untreated samples, after substracting the OD of NMS (background), then multiplying by 100. Antibodies with AI values less than 30%, between 30 and 50%, and above 50% were considered low-avidity, intermediate-avidity, and high-avidity, respectively [27]

### Titration assay reveals adjuvanted cd-HAP formulations elicit strong and specific humoral immune response

**Fig. 5:**
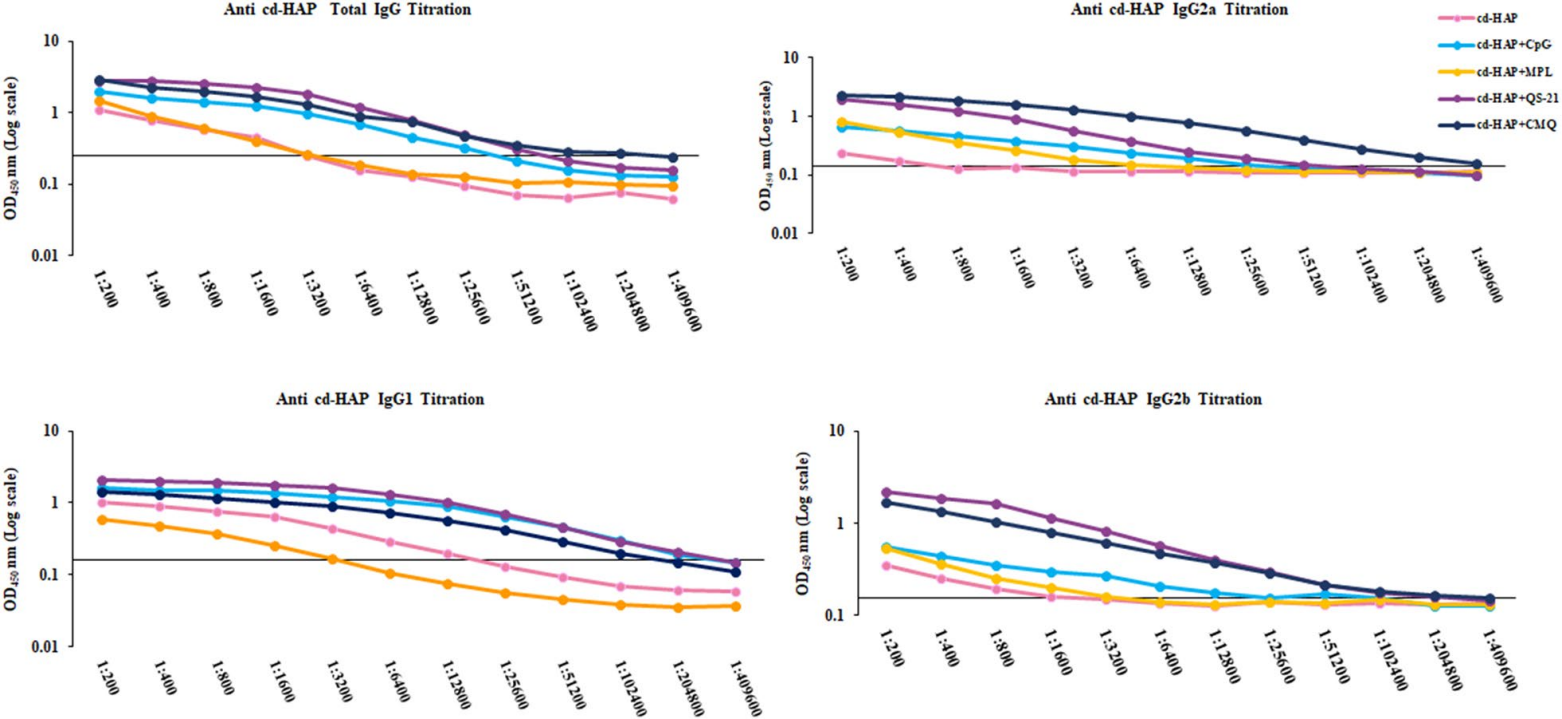
Endpoint titration of anti-cd-HAP IgG, IgG1, IgG2a, and IgG2b antibodies in different vaccine groups. Mouse sera from groups 1–5 were analysed by ELISA with dilutions ranging from 1:200 to 1:409,600. The titers were determined as the last dilution with an OD_450_ nm above the corresponding cut-off value for each antibody subclass: IgG (0.26), IgG1 (0.2), IgG2a (0.16), and IgG2b (0.15). The cut-off values are indicated with a horizontal line. The highest endpoint titers of anti-cd-HAP total IgG, IgG2a, and IgG2b were observed in group 5 (cd-HAP/CMQ), while the lowest endpoint titers for IgG were detected in groups 1 (cd-HAP alone) and 3 (cd-HAP/MPL)

The end-point titers of anti-cd-HAP IgG, IgG1, IgG2a, and IgG2b antibodies were measured by ELISA on day 38 after the first immunization in all five immunized mouse groups. The results showed that the highest end-point titers of anti-cd-HAP total IgG, IgG2a, and IgG2b antibodies were observed in the mouse Group 5 (cd-HAP/CMQ) (204,800 for IgG, and 409,600 for IgG2a and IgG2b, Fig. 5) with the same end-point titer of IgG2b for group 4 (cd-HAP/QS-21). In contrast, the lowest end-point titers of IgG (3200) was detected in the mouse Groups 1 (cd-HAP antigen alone) and 3 (cd-HAP/MPL). Among immunized mice, group 1, which received the cd-HAP antigen alone, had the lowest end-point titers for IgG2a (400) and IgG2b (1600) antibodies. The highest end-point titer of IgG1 was observed in the mouse Groups 2 and 4 (409,600) followed by Group 5 (102,400). However, the lowest end-point titer of IgG1 was detected in mouse groups receiving cd-HAP/MPL (3200) and cd-HAP (25,600) (Fig. 5).

### Cytokine production analysis reveals potent Th1 immune response induced by cd-HAP formulated with CMQ adjuvants

**Fig. 6:**
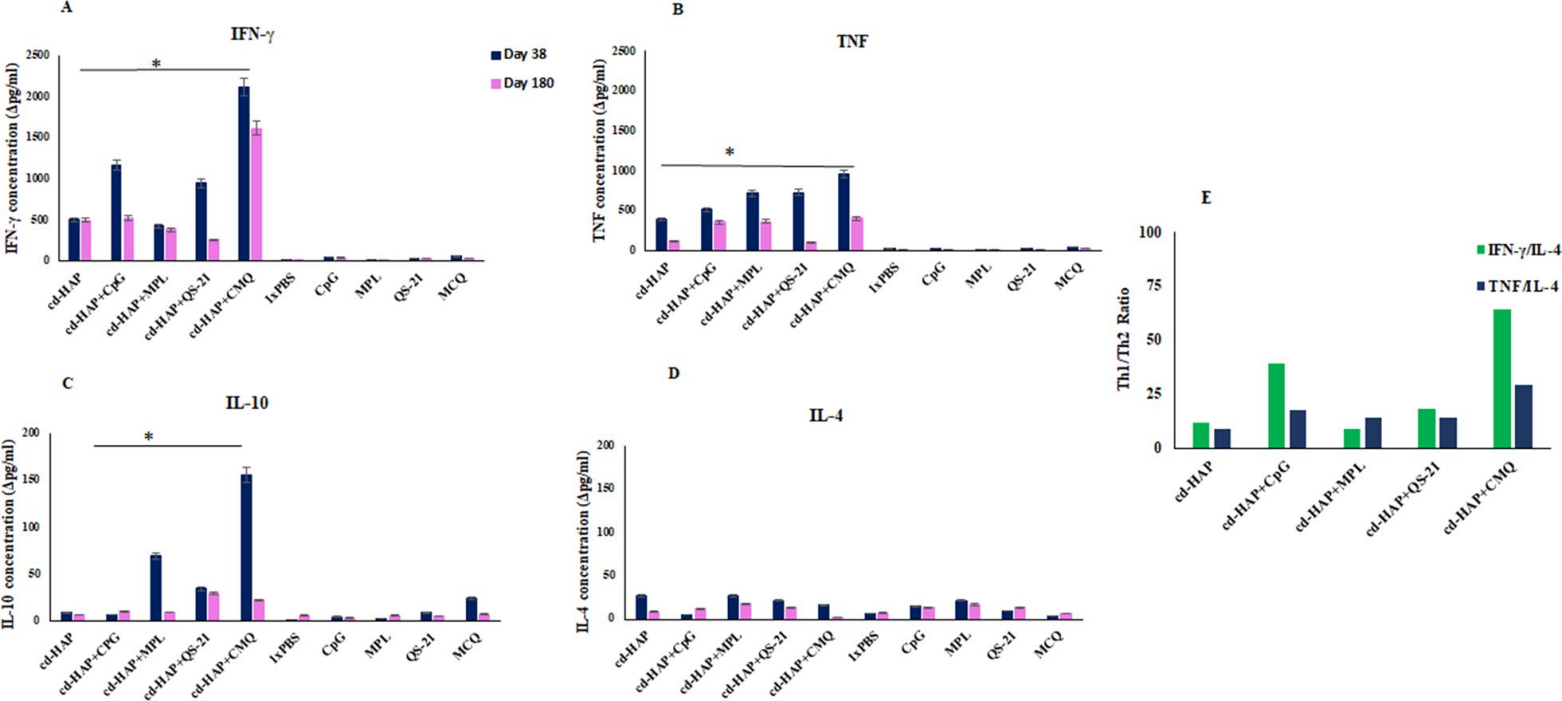
Cytokine responses were evaluated in immunized and control groups (3 randomly selected mice from each group at each time point) on days 38 and 180 after primary immunization. Mean cytokine responses for IFN-γ, TNF, IL-10, and IL-4 in the presence of cd-HAP antigen are shown in (**A**–**D**), respectively. To remove the backgrounds, the concentration of each cytokine in negative control (by media) was subtracted from the concentration of the same cytokine of test group stimulated by antigen and delta IFN-γ, TNF, IL-10, and IL-4 were presented in the figures. One-way ANOVA analysis showed significant mean difference for the level of IFN-γ, TNF, and IL-10 cytokines between vaccine groups 1–5. *P < 0.0001. The bars and error bars show the mean concentrations (pg/ml) of relevant cytokines and standard deviations (SD), respectively. Cytokine levels were shown on day 180 compared to day 38. **E** The IFN-γ/IL-4 and TNF/IL-4 ratio values, as an indicator of Th1/Th2 immune response, was calculated for each group on day 38

Cytokine production was measured in the supernatants of in vitro splenocyte cultures stimulated with cd-HAP antigen in immunized mouse groups (3 randomly selected mice from each group at each time point) on days 38 and 180 after the primary immunization. Analysis of cytokine profiles revealed significant levels of IFN-γ in all immunized Groups 1–5, compared to the mouse control groups on both days 38 and 180 after the primary immunization (P < 0.0001, One-way ANOVA, Fig. 6A). The highest levels of IFN-γ production was detected in immunized mouse Group 5 (cd-HAP/CMQ = 2140 pg/ml), and the lowest levels of IFN-γ production were detected in mouse Group 3 (cd-HAP/MPL = 453.9 pg/ml) and non-adjuvanted mouse Group 1 (cd-HAP = 538.76 pg/ml) on days 38 of the primary immunization (P < 0.0001, One-way ANOVA, Fig. 6A). Furthermore, among single adjuvanted immunized Groups 2–4, the level of IFN-γ in Group 2 receiving antigen plus CpG was significantly higher than that in Groups 3 and 4 receiving antigen plus MPL and QS-21, respectively, on both days 38 and 180 after the primary immunization (P < 0.0001, Bonferroni post hoc test, Fig. 6A). The level of IFN-γ significantly decreased in immunized mouse Groups 2 (cd-HAP/CpG), 4 (cd-HAP/QS-21), and 5 (cd-HAP/CMQ) on day 180 after the primary immunization (P < 0.05, paired sample t-test, Fig. 6A), but the highest level of IFN-γ was still observed in mouse Group 5 (cd-HAP/CMQ) relative to other immunized mouse groups on day 180 (1639.61 pg/ml, P < 0.0001, One-way ANOVA, Fig. 6A).

Analysis of TNF production on day 38 after primary immunization revealed a significant difference between mouse groups 1–5 receiving different formulations of cd-HAP antigen and the control groups 6–10 (P < 0.0001, One-way ANOVA, Fig. 6B). The highest and lowest levels of TNF were produced by mouse Group 5 (cd-HAP/CMQ: 968.6 and 416 pg/ml) and Group 1 (cd-HAP: 407.2 and 134.3 pg/ml) on days 38 and 180 of the primary immunization, respectively (P < 0.05, One-way ANOVA, Fig. 6B). The level of TNF in mouse Group 5 (cd-HAP/CMQ: 968.6 pg/ml) was significantly higher than single adjuvanted immunized mouse Groups 2–4 (cd-HAP/MPL: 735.55 pg/ml, cd-HAP/CpG 534.36 pg/ml, and cd-HAP/QS-21 758.09 pg/ml) on day 38 of the primary immunization (P < 0.05, Bonferroni post hoc test, Fig. 6B). Comparing the level of TNF production on day 180, in all immunized mouse Groups 1–5 revealed that the highest level of TNF was still produced by the splenocytes of the mice receiving cd-HAP with CMQ adjuvants (Fig. 6B).

IL-10 production, as a regulatory cytokine, was low (< 200 pg/ml) in all immunized mice (1–5) and control (6–10) groups (< 50 pg/ml) on days 38 and 180 of the primary immunization. However, there was a significant difference in the level of IL-10 between immunized mouse Groups 1–5 on day 38 after primary immunization (mean IL-10 concentration: 10–172 pg/ml) (P < 0.0001, One-way ANOVA, Fig. 6C). Regarding the analysis of IL-4 secretion as a Th2 type response, low levels of IL-4 with no significant difference were detected in different immunized (1–5) and control (6–10) mouse groups at both time points, on days 38 and 180 (Fig. 6D).

The ratios of IFN-γ/IL-4 and TNF/IL-4 were calculated to assess the ratio of Th1 to Th2 immune responses induced by different formulations of antigen/adjuvant(s). Among immunized mouse groups, the highest IFN-γ/IL-4 ratio was observed in mice immunized with cd-HAP formulated with the combination of adjuvants (CMQ) on day 38 after the primary immunization, indicating a predominant Th1 response. Among the mice receiving cd-HAP antigen formulated with single adjuvants, the highest ratio of IFN-γ/IL-4 and TNF/IL-4 was observed in Group 2 receiving cd-HAP/CpG formulation. In contrast, these ratios in mice immunized with cd-HAP/MPL and cd-HAP alone showed the lowest ratio of Th1/Th2 immune responses among immunized mouse groups. The ratios of TNF/IL-4 showed a similar trend, with the highest ratio observed in the cd-HAP/CMQ group on day 38, followed by cd-HAP/CpG. These results suggest that the combination of cd-HAP with CMQ adjuvants is a potent inducer of Th1 immune responses.

### Eliciting anti-cd-HAP antibodies in mice reduces *P. falciparum* oocyst formation in *An. stephensi* midgut using the SMFA

**Fig. 7:**
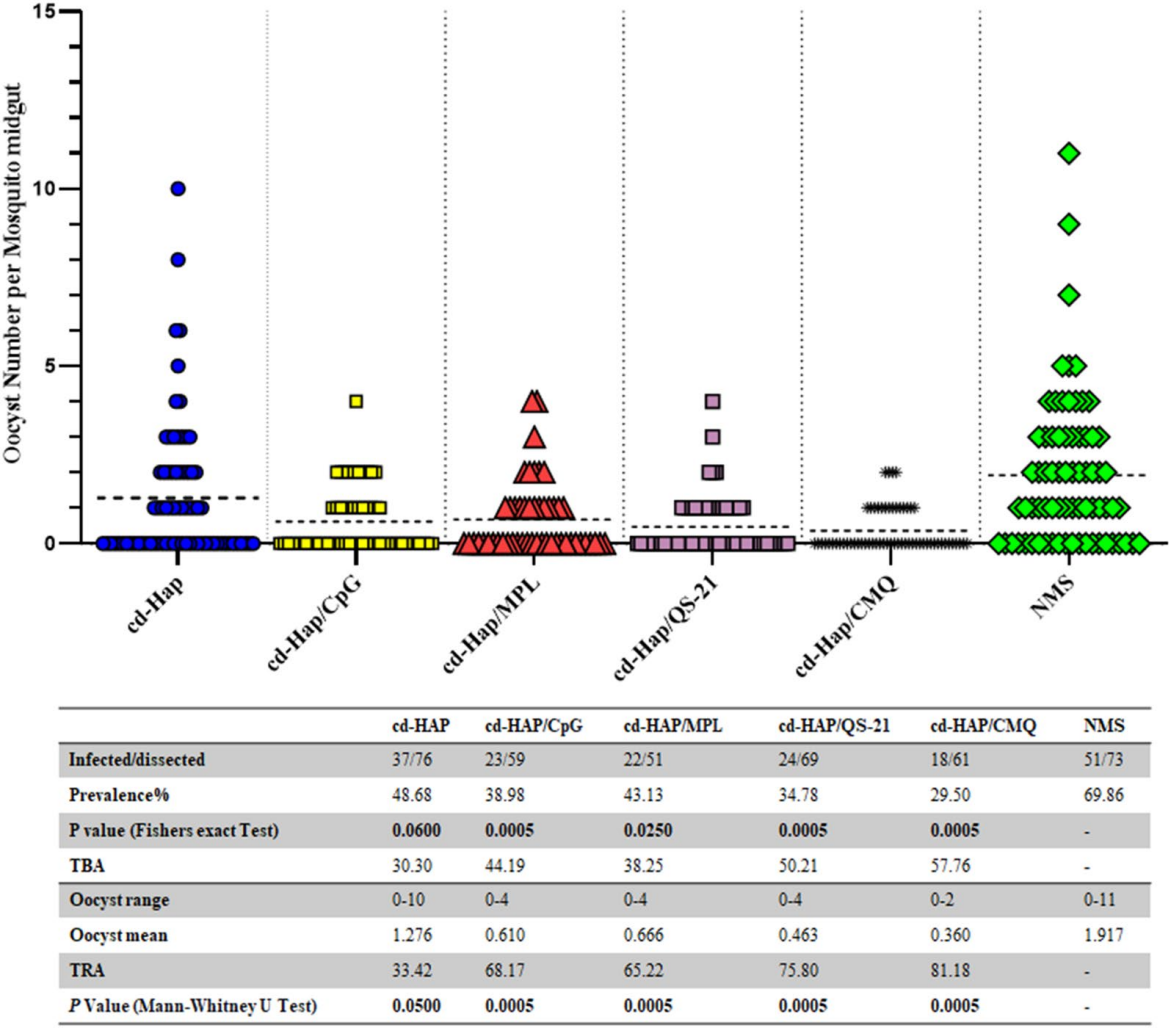
Inhibition of *P. falciparum* NF54 parasite development by anti-cd-HAP polyclonal antibodies in *An. stephensi* mosquitoes. Mouse sera from different vaccine groups (groups 1 to 5), collected on day 38 after the first immunization, were pooled (n = 9) and combined with mature *P. falciparum* NF54 cultured gametocytes. The mixture was then fed to *An. stephensi* mosquitoes (n = 50/cup) in standard membrane feeding assays (SMFAs). Negative control was conducted using pooled normal mouse serum (NMS) (n = 30 randomly selected from 160 female BALB/c mice before immunization). Oocyst counts, which indicate the successful development of *P. falciparum* in *An. stephensi* mosquitoes, were recorded by dissecting the mosquitoes’ midguts on days 9 to 10 after feeding. Two separate membrane feeds were done for each vaccine group (groups 1 to 5) and the oocyst counts were pooled for statistical analysis. The dots on the graph represent the oocyst count distribution. The table provides information on the prevalence of infected mosquitoes in each group, the range and mean number of oocysts, the percent inhibition relative to the NMS control group, and multiple comparisons between different vaccine groups and the NMS control group. Statistical analysis was conducted using the Mann–Whitney U test and Fisher’s exact test to estimate the differences in infection intensity and prevalence, respectively. All P values were adjusted with Bonferroni correction analysis. P < 0.05 was considered statistically significant and shown in bold

The present study aimed to investigate the inhibitory effects of anti-cd-HAP polyclonal antibodies elicited from immunized mouse groups on the development of *P. falciparum* NF54 parasites in *An. stephensi* mosquitoes using the SMFA. The pooled sera of the immunized mouse Groups 1–5, which received cd-HAP antigen alone or in combination with different adjuvants, including cd-HAP, cd-HAP/CpG, cd-HAP/MPL, cd-HAP/QS-21, and cd-HAP/CMQ, respectively, were distinctly tested for their inhibitory activity. Similar oocyst intensity and infection prevalence were observed in mosquitoes fed by blood meal containing normal mouse serum (NMS) or serum from control groups 6–10 (Additional file 1: Fig. S1). This observation indicates that the collected sera from adjuvanted control groups had no inhibitory effect on parasite development in the mosquito. The results revealed that the induced antibodies in different mouse groups had varying levels of transmission-reducing activity (TRA) of *P. falciparum* infection in *An. stephensi* mosquitoes. The mouse sera collected from all adjuvanted immunized groups showed significant TRA (G2: 68.17%, G3: 65.22%, G4: 75.80%, and G5: 81.18%) in comparison to the control group (adjusted *P* < 0.05, Mann–Whitney test, Fig. 7). Interestingly, administration of cd-HAP antigen with CMQ (G5) showed significant TRA relative to G1 immunized with antigen alone (adjusted* P* = 0.015). In particular, the polyclonal anti-cd-HAP antibodies from the mouse Group 5 (cd-HAP/CMQ) exhibited the highest TRA (81.18%) (Fig. 7). Furthermore, transmission-blocking activity (TBA) of sera in different mouse groups revealed that among the immunized groups, the highest and lowest TBA was detected in mouse Groups 5 (cd-HAP/CMQ, 57.76%) and 1 (cd-HAP, 30.30%), respectively. The infection rate in mosquitoes fed by blood meal containing NMS was 69.86%. The proportion of infected mosquitoes in all adjuvanted immunized groups was significantly different from the control group fed by NMS (adjusted* P* < 0.05, Fisher’s exact test, Fig. 7).

## Discussion

According to WHO malaria guidance (2023), global efforts must be made to control and eliminate malaria in accordance with the Global Technical Strategy for Malaria 2016–2030 (GTS). Conferring to GTS, malaria cases and deaths must be reduced by 90% by 2030. To this aim, vaccine development is indispensable, and since 2021, the WHO recommended RTS,S as the only available vaccine, to reduce the death and severe *P. falciparum* malaria in moderate to high transmission areas. However, for elimination and eradication strategies, TBVs are in priority to interrupt transmission. In this regard, the current study was designed to evaluate a new fusion protein (cd-HAP), as a TBV candidate antigen.

Antibody responses induced by recombinant proteins must be able to recognize the native form of the antigen on the parasite in order to be effective [32]. Interestingly, all positive sera for PfGCS1, were able to recognize the new fusion cd-HAP antigen, implying that native epitopes of PfGCS1 are recognized by cd-HAP antigen. Furthermore, analysis of the profile of IgG subclasses in the current study, demonstrated the high levels of anti-cd-HAP IgG3, IgG1, and IgG2 antibodies in response to malaria infection that is consistent with previous studies conducted with other antigens [33–35]. IgG1 and IgG3 antibodies are known to activate complement-mediated lysis of the parasite, and they also promote phagocytosis and killing of the parasite by macrophages and other phagocytic cells [36, 37], even in the midgut of mosquitoes [38, 39]. In the current investigation, the high level of naturally acquired anti-cd-HAP IgG1 and IgG3 antibodies emphasizes that the new fusion protein has the common epitopes with the native PfGCS1 for these opsonizing antibodies. Hence, they might act by opsonizing of microgametes in the midgut of *Anopheles*, helping for transmission inhibition.

For a TBV candidate antigen to be effective, it should have the capacity to stimulate the production of transmission-blocking antibodies [40]. Previous studies have shown that the antibodies against antigens of gametocytes are associated with reduced gametocyte density in malaria patients [41]. The results of the current study showed that cd-HAP antigen formulated with a combination of adjuvants (CMQ) induced the highest level (mean OD_450_nm: 2.83) and titer (204,800) of anti-cd-HAP IgG antibodies with the highest transmission-reducing activity 81.18%, suggesting that this formulation is more potent than single adjuvant formulations (65.22–75.80%) for transmission-reducing activity (P < 0.05). This result is consistent with the previous study with Pfs25/Pvs25 antigens that have shown potent humoral immunity is required for successful malaria transmission-blocking immunity [42].

Analysis of the IgG subclasses revealed the highest levels (OD_450_nm: 2.25) and titer (409,600) of anti-cd-HAP IgG2a antibodies in the mice receiving cd-HAP antigen formulated with a combination of adjuvants (CMQ), suggesting that this formulation may favor the induction of potent Th1 immune responses. The high levels of anti-cd-HAP IgG2a antibodies in mice receiving the cd-HAP antigen with the combination of adjuvants (CMQ), is consistent with the findings of other studies which have reported that the use of some combination of adjuvants can result in improving the immunogenicity compared to the single adjuvants [18, 43, 44]. Previous studies revealed that Th1-type antibodies (IgG2a and IgG2b) play a main role in transmission immunity by opsonizing the gametes, followed by phagocytosis by leukocytes [38, 39]. The obtained results revealed that this fusion protein formulated with CMQ adjuvants can elicit cytophilic anti-cd-HAP IgG antibodies (IgG2a and IgG2b) that may lead to the opsonizing of microgametes in mosquito host, followed by phagocytosis. Besides, all mouse IgG antibodies except IgG1 have functional activity via complement activation [45]. Regarding Pfs230 as another TBV candidate, it has been shown that a strong Th1-type immune response with high levels of IgG2a and IgG2b has functional activity due to complement activation, leading to gamete lysis [46, 47]. The observed high levels of IgG2a and IgG2b in groups with higher transmission-blocking activity in this study may suggest a potential role of complement activation in transmission-blocking immunity induced by different formulations of cd-HAP antigen with adjuvants, although further investigation is needed to confirm this conclusion.

It was interesting that mice immunized with the cd-HAP antigen plus CMQ adjuvants exhibited higher avidity indices for anti-cd-HAP IgG (49%), IgG2a (60%), and IgG2b (44%) antibodies compared to mice immunized with the cd-HAP antigen alone or in combination with single adjuvants. So, this formulation may support the maturation of immune responses and the production of high-avidity antibodies. Furthermore, the obtained results indicate that mouse group 5 with a higher avidity index of IgG, IgG2a, and IgG2b antibodies had more transmission-blocking activity than other groups. This finding is in accordance with a previous study that investigated the blocking activity of antibodies induced against different formulations of Pfs25 antigen, and revealed that mouse groups with high avidity antibodies had more transmission-blocking activity [48].

There are pieces of evidence for cell-mediated immunity to be effective in reducing malaria transmission. Some of the released cytokines, such as IFN-γ, caused by cellular immunity can help with antibody production and also antibody switching that can help in inducing the cytophilic antibodies [49]. As mentioned above, cytophilic antibodies are effective components in transmission-blocking immunity, through the effect on gametes in the mosquito midgut. In addition, it has been shown that some cytokines such as IFN-γ and TNF can reduce the infectivity of gametocytes [50, 51].

## Conclusion

This study showed that the new fusion protein, cd-HAP antigen, has an appropriate conformation with common epitopes with native antigens on the male gametocytes. cd-HAP antigen formulated with a combination of adjuvants (CMQ) can induce potent Th1 immune responses with inhibitory antibodies for oocyst formation, implying that this fusion protein acts as an appropriate TBV candidate. The obtained findings also underscore the critical role of adjuvants in augmenting the efficacy of the immune response elicited by the antigen and demonstrated 81.18% transmission-reducing activity. These results with other similar results of the previous studies [18, 19, 52] contribute to the growing body of evidence that supports the use of vaccines in interrupting malaria disease transmission. In addition, the results emphasize the importance of adjuvant selection in optimizing antigen immunogenicity and efficacy. To increase the blocking efficacy of this formulation, the addition of other TBV candidate antigens and the evaluation of the safety and efficacy of that are suggested.

### Supplementary Information

**Additional file 1.** Oocyst number development in normal mouse sera (NMS) compared to control groups 6-10 using standard membrane feeding assay (SMFA). Mouse sera from naïve mice or different control groups 6-10, collected on day 38 after the first immunization combined with mature *P. falciparum* NF54 cultured gametocytes. The mixture was then fed to *An. stephensi* mosquitoes in SMFA. Oocyst counts were recorded by dissecting the mosquitoes’ midguts on days 8 to 10 after feeding. The dots on the graph represent the oocyst count distribution, and the horizontal dashed lines represent for oocysts mean. No significant mean oocyst difference was observed between different control groups and NMS (P > 0.05, Mann–Whitney U test).

## Data Availability

All data will be available by the corresponding author (Dr. Abouie Mehrizi, Email: abouei@gmail.com) under request.
