# Supplementary figures and images for: Evaluation of a new fusion antigen, cd loop and HAP2-GCS1 domain (cd-HAP) of Plasmodium falciparum Generative Cell Specific 1 antigen formulated with various adjuvants, as a transmission blocking vaccine
Source: Malar J. 2023 Dec 9;22:374. doi: 10.1186/s12936-023-04798-7 (PMC10710725; doi:10.1186/s12936-023-04798-7)

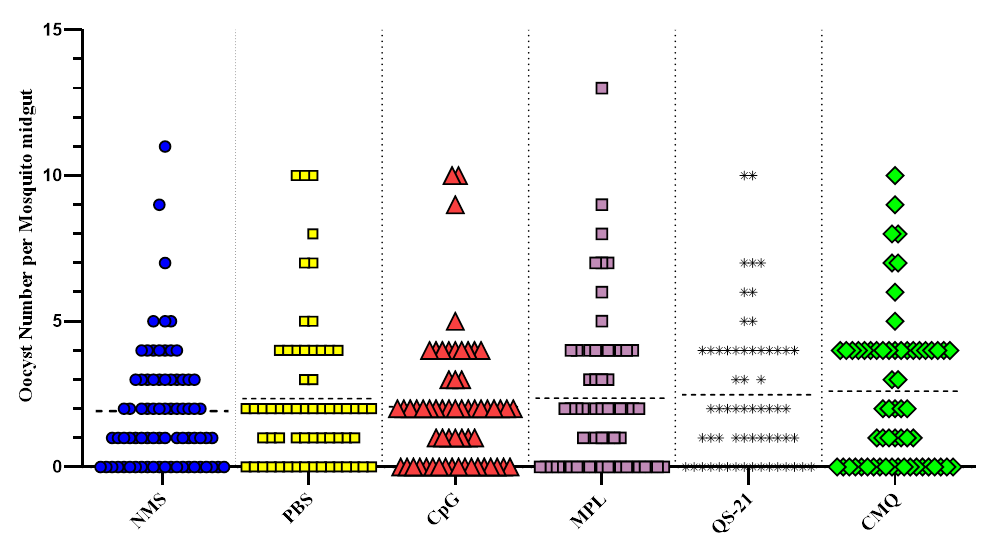

Supplement: Supplementary file 1 — Additional file 1. Oocyst number development in normal mouse sera (NMS) compared to control groups 6-10 using standard membrane feeding assay (SMFA). Mouse sera from naïve mice or different control groups 6-10, collected on day 38 after the first immunization combined with mature P. falciparum NF54 cultured gametocytes. The mixture was then fed to An. stephensi mosquitoes in SMFA. Oocyst counts were recorded by dissecting the mosquitoes’ midguts on days 8 to 10 after feeding. The dots on the graph represent the oocyst count distribution, and the horizontal dashed lines represent for oocysts mean. No significant mean oocyst difference was observed between different control groups and NMS (P > 0.05, Mann–Whitney U test). [file 12936_2023_4798_MOESM1_ESM.tif]
